# Towards the Development of a Sensor Educational Toolkit to Support Community and Citizen Science

**DOI:** 10.3390/s22072543

**Published:** 2022-03-26

**Authors:** Ashley Collier-Oxandale, Vasileios Papapostolou, Brandon Feenstra, Berj Der Boghossian, Andrea Polidori

**Affiliations:** South Coast Air Quality Management District, Air Quality Sensor Performance Evaluation Center (AQ-SPEC), Diamond Bar, CA 91765, USA; vpapapostolou@aqmd.gov (V.P.); bfeenstra@aqmd.gov (B.F.); bderboghossian@aqmd.gov (B.D.B.); apolidori@aqmd.gov (A.P.)

**Keywords:** air quality, sensors, citizen science, community monitoring, environmental justice, education and outreach

## Abstract

As air quality sensors increasingly become commercially available, a deeper consideration of their usability and usefulness is needed to ensure effective application by the public. Much of the research related to sensors has focused on data quality and potential applications. While this information is important, a greater understanding of users’ experience with sensors would provide complementary information. Under a U.S. EPA-funded Science to Achieve Results grant awarded to the South Coast Air Quality Management District in California, titled “Engage, Educate, and Empower California Communities on the Use and Applications of Low-Cost Air Monitoring Sensors”, approximately 400 air quality sensors were deployed with 14 California communities. These communities received sensors and training, and they participated in workshops. Widely varying levels of sensor installation and engagement were observed across the 14 communities. However, despite differences between communities (in terms of participation, demographics, and socioeconomic factors), many participants offered similar feedback on the barriers to sensor use and strategies leading to successful sensor use. Here, we assess sensor use and participant feedback, as well as discuss the development of an educational toolkit titled “Community in Action: A Comprehensive Toolkit on Air Quality Sensors”. This toolkit can be leveraged by future community and citizen science projects to develop networks designed to collect air quality information that can help reduce exposure to and the emissions of pollutants, leading to improved environmental and public health.

## 1. Introduction

Members of the general public are increasingly becoming engaged in air quality research. Concerns over threats to public and environmental health are typical motivating factors, while the continual advancement of air quality sensors helps to facilitate this participation in community and citizen science (CCS) [1]. Typically, consumer-grade air quality sensor devices cost 100–3000 USD each and measure one or more pollutants, as well as environmental parameters. However, the individual or OEM (original equipment manufacturer) sensor used in these devices often costs far less and may be incorporated into custom devices. Additionally, sensors are typically small or portable and do not require the same rigorous training and maintenance as conventional air quality monitoring instruments. As a result of these trends, the number of sensor devices and the number of projects using them continues to grow [2]. Whether this technology contributes to improved air quality depends on more than its availability.

### 1.1. Current Understanding of Air Quality Sensing Technology

Much of the research into air quality sensors has focused on sensor performance and potential uses for sensor data. This focus likely stems from ongoing concerns over data quality and the technology’s reliability [3]. Studies have examined sensor performance in laboratory settings and during field deployments [4,5,6,7,8,9]. Researchers have explored different approaches to sensor calibration [10,11,12]. Sensors have also been studied in the context of a variety of different applications, including reducing personal exposure, monitoring near sources, and increasing the spatial resolution of data [13,14,15,16]. 

In support of sensor performance assessments, testing protocols for evaluating sensors have been developed by the U.S. Environmental Protection Agency (EPA), the European Union Joint Research Centre (JRC), and the South Coast Air Quality Management District (South Coast AQMD) [17,18,19]. The Air Quality Sensor Performance Evaluation Center (AQ-SPEC), established at the South Coast AQMD in 2014, conducts evaluations of commercially available sensors and posts the reports online for use by the public (http://www.aqmd.gov/aq-spec, accessed on 15 February 2022). As our understanding of sensor capabilities increases, this information would be complemented by a better understanding of how to facilitate successful sensor use by citizen scientists and how to appropriately engage, educate, and empower emerging community air monitoring networks. 

### 1.2. Use of Air Quality Sensors by Members of the Public

Sensor operation, including everything from siting to quality control (QC) procedures, is essential as it can impact the collection of high-quality useful data. However, research on sensor use by members of the public is limited. One review highlights the need to study how to encourage successful collaborations among government, academia, and communities and ways to communicate data and results [20]. Another group identified a better understanding of how individuals and communities would like to use sensors and the data, ways to encourage effective sensor use, a consideration of the ethical implications, and ways to ensure effective communication and interpretation of data as key research needs [21]. However, from a user perspective, there is also little information on practical issues such as the siting, installation, proper operation, and maintenance of sensors by members of the public. Two studies explored questions related to sensor siting; however, in both studies, the researchers installed the sensors as opposed to community members [22,23]. 

A few studies have leveraged participatory or transformation design, user experience studies, and the study of human–computer interactions (HCIs) to explore or improve the design of sensors and associated data platforms [24,25,26,27]. Another highlighted the importance of making knowledge portals and centralized data platforms available to participants, as well as discussing the roles environmental agencies can play [28]. Some studies used participant feedback to improve the function of sensors and data platforms [25,27]. One study found that prompting active participation through the data interface caused participation and engagement to remain high throughout the project [26]. While these examples offer valuable insights, these studies often rely on prototype sensors and relatively few participants or a single community. A thorough examination of the efficacy of commercially available sensors throughout all phases of a typical community-based project and when used by more participants and different communities is needed. 

A better understanding of this aspect of sensor use would enable the development of appropriate guidebooks and resources to enhance community-based participatory air monitoring campaigns. Highlighting the value of a more comprehensive understanding of this technology, a study comparing the impact of a project on knowledge and attitudes using sensors in three communities in Europe observed different results for those directly participating versus the broader regional population. Researchers found that the projects resulted in benefits for the participating individuals in all regions; however, each project’s impact at a regional population level was mixed in terms of success [29]. The researchers believe these results were driven by differences in established local environmental organizations and the data communication in each region [29]. Although the same technology was used, the project’s impact on public knowledge and attitudes varied due to the surrounding context and implementation, which highlights the importance of studying the efficacy of this technology in real-world applications.

### 1.3. Previous Community or Citizen Science Air Monitoring Projects

As the use of air quality sensors scales up, leveraging knowledge from different disciplines may help shape the applications of this technology. For example, conventional air quality monitoring research can help improve data quality. The fields of citizen science and community-based participatory research can facilitate effective partnerships and projects [30,31]. The fields of data science, geospatial analysis, and data communication can be leveraged to manage the large datasets and transform these into meaningful information to be displayed on data dashboards [32,33,34]. Lastly, aspects of computer science, such as HCI and participatory design, as described previously, may help shape the technology itself. 

In particular, community-based air quality monitoring projects that rely on conventional monitoring methods and instruments may hold valuable lessons for projects using sensors. As an example, Drift Catcher involves members of the public monitoring for pesticides using relatively complex equipment [35]. An assessment of Drift Catcher projects found that community partners with strong organizational capacity were better equipped to adhere to sampling protocols, as well as to understand and make use of the data, resulting in more successful projects [35]. Other projects may use combined teams of community members and researchers to conduct the sampling and ensure the collection of high-quality, relevant data [36] or combine air quality data collected by researchers with observations collected by community members [37]. However, these projects often involve sampling by community members and then processing of those samples by laboratory methods, which can limit the number of samples and scope of the project [36,38].

In addition to considering how projects are conducted, the outcomes of projects using conventional methods may inform possible objectives or measures of success for community or citizen science sensor projects. A review of these types of projects shared the following list of positive impacts: increased awareness of air quality issues, new data and forecasts made available, funding and support for equipment replacement programs, the establishment of new monitoring sites, and locally relevant procedures to help reduce exposures to certain pollutants [36,37,39]. Although air quality sensors are a novel technology, a few studies have noted the potential impacts and outcomes of sensor use. For example, portable sensors were provided to youth to measure their personal exposure to air pollution, which resulted in enhanced environmental health literacy [40]. Another study, examining how the use of a sensor influenced perceptions and behaviors, found that the hands-on use of a sensor can increase knowledge of air pollutants and how to mitigate the risks associated with those pollutants [41]. In one community, data from a network of sensors are being used for local decision making. The data are categorized using a scale similar to the Air Quality Index (AQI) that provides public health recommendations and enables local schools to use this information to make decisions regarding outdoor activities [42]. There are also examples of sensors being used as “environmental health thermometers” for real-time decision making such as validating or invalidating suspected exposures to local sources [43]. 

Continued research into the perspectives and experiences of sensor users will enable the development of best practices and necessary supplemental resources. Furthermore, research must address this technology specifically as it differs from conventional air quality monitoring in key ways. Commercial sensor devices may be purchased, installed, and maintained by community members, with data streaming automatically to publicly available online platforms for analysis and visualization, requiring minimal involvement by researchers. Hence, a better understanding of sensor use in the context of CCS projects will complement existing knowledge and enable the collection of high-quality, useful data.

### 1.4. Science to Achieve Results 

In this paper, we present results from a South Coast AQMD project, entitled “Engage, Educate, and Empower California Communities on the Use and Applications of Low-Cost Air Monitoring Sensors” funded by the U.S. EPA Science to Achieve Results (STAR) Grant program. These results include an analysis of levels of participation and engagement in the project, a summary of issues with and barriers to sensor use, feedback on the usefulness of sensors to participants, and overall lessons learned. A key outcome of this project is the development of an educational toolkit titled “Community in Action: A Comprehensive Toolkit on Air Quality Sensors” (http://www.aqmd.gov/aq-spec/special-projects/star-grant, accessed on 15 February 2022), which was informed and shaped by the work performed within the STAR Grant. This toolkit is intended to complement currently available resources, such as the Citizen Science Toolbox offered by the U.S. EPA [44] and previously developed air sensor guidebooks [45,46,47]. The resources in the toolkit have the advantage of leveraging the results of a large-scale (nearly 400 sensor units deployed and over 350 community members engaged), long-term (with sensor deployments lasting up to three years) project conducted with 14 different California communities. The scope and scale, in particular, make this project unique compared to similar efforts [42,48]. The results of this project and the development of the educational toolkit are discussed to provide future CCS air quality projects and programs with the knowledge necessary to use and apply sensing technology and available resources to effectively and efficaciously engage, educate, and empower the public in air monitoring efforts.

## 2. Materials and Methods

The objective of this project was to determine how to provide communities with the necessary knowledge to appropriately select, use, and maintain air quality sensors and to correctly interpret the data collected by large sensor networks. The project was driven by the following four specific aims: (1) develop new methodologies to educate and engage communities on the use and applications of “low-cost” sensors, (2) conduct testing to characterize the performance of commercially available “low-cost” sensors and to identify candidates for field deployment, (3) deploy the selected sensors in California communities, and interpret the collected data, and (4) communicate the lessons learned to the public through a series of outreach activities [49]. This paper focuses on the community engagement aspect of this project in aims 1, 3, and 4. 

### 2.1. Project Overview

Initial project activities involved recruiting communities, establishing partnerships, and meeting with communities to introduce the project and discuss local air quality concerns. Project leads both established new and leveraged existing relationships with community group leaders to recruit project participants. Preliminary discussions with local partners revealed consistent concerns about particulate matter (PM) in many participating communities (Figure S1); hence, this pollutant became the focus. Under the AQ-SPEC program at South Coast AQMD, the performance of commercially available sensors was evaluated to identify suitable candidate devices [6,7]. At the time of sensor selection, AQ-SPEC had tested around 15 air quality sensors, one of which was the PA-II sensor by PurpleAir. The PA-II uses optical particle counting technology to estimate PM_1.0_, PM_2.5_, and PM_10_ mass concentrations. The sensor collects near-ambient weather data (temperature, humidity, and pressure) and is connected to available Wi-Fi to automatically stream and display data on a real-time map (www.purpleair.com/map, accessed on 15 February 2022). This sensor was determined to be well suited for use in the STAR Grant project based on several factors. The cost of the sensor is approximately 200 USD per device, and this relatively low cost per device allowed for the distribution of a larger number of sensors than initially proposed. Another factor was the free and open-access nature of the data, which allowed both sensor hosts and other community members to easily engage with the data. Some sensors require data hosting or subscription costs as the infrastructure to maintain data archives and support data access can be costly. However, PurpleAir provides this service free of charge to sensor users and the larger public. This open-access data model has limitations as well, for example, features such as custom alerts or notifications may not be available. This sensor also performed relatively well in AQ-SPEC field and laboratory evaluations for PM_2.5_ compared to other similar sensors commercially available at the time [6]. The average *R*^2^ value across the triplicate was 0.95, and the average mean absolute error across the triplicate was 6.8 µg/m^3^ [6]. An excerpt from the AQ-SPEC field evaluation report is available in the Supplementary Materials (Figure S2). In these evaluations, the performance for PM_1.0_ was comparable to PM_2.5_; however, the sensor did not perform as well for PM_10_. Additional multi-sensor units (AQY v0.5 units by Aeroqual) were added in some communities to provide supplemental air quality data; this was undertaken at the request of the participating communities. These supplemental sensors were also thoroughly tested/evaluated by the AQ-SPEC Program. These sensor units include sensors for PM_2.5_, nitrogen dioxide (NO_2_), and ozone (O_3_). For most of the analysis (undertaken by the project leads at the South Coast AQMD), data were used as provided by PurpleAir, with no additional corrections applied. This approach was used because project leads wanted to use the same data viewed and accessed by community members.

Sensors (the PurpleAir PA-II sensors) were then distributed at training workshops to those community members who decided to participate in this research project. Within this project, the researchers primarily deployed sensors in a one-to-one relationship between sensor and community member. This was made possible due to the low cost of the sensor selected and was dependent on volunteers within the community. Throughout the sensor deployment period, multiple community workshops were conducted with all participating communities to discuss the use and operation of the sensors, the usefulness and quality of the collected data, and participants’ air quality observations. 

### 2.2. Participating Communities 

Partnerships were established initially with six groups (e.g., community-based organizations and local air quality management agencies), resulting in the implementation of the project in eight communities. Note that many of these communities were selected on the basis of the presence of local environmental justice issues. As news of the project spread, additional communities expressed strong interest in participating. South Coast AQMD was able to expand upon the original grant and collaborate with an additional six communities, bringing the total to 14. Some communities were provided with funding to support their participation, while others participated on a volunteer basis. Accommodating the involvement of some communities on a volunteer basis facilitated the engagement of a wider variety of communities in terms of demographic makeup, resulting in more broadly applicable results (Figure S3). 

Here and throughout, communities are identified using letters to provide anonymity. In terms of racial/ethnicity distributions, the participating communities also varied and included areas where residents were majority or in part Hispanic/Latino/Spanish Origin, Asian, Hawaiian Native/Pacific Islander, African American/Black, or white. The communities varied geographically, in terms of nearby land use, and in terms of local environmental justice issues. Eight communities were in southern California, two in central California, and two in northern California. Community locations included rural inland areas, coastal areas, and high-density urban/suburban areas. Nearby land use ranged from major roadways and other types of transportation to industrial, residential, agricultural, and more. 

### 2.3. Engagement with Communities and Information Collected 

Project leads conducted a series of workshops (i.e., introduction to the project, pre-sensor deployment, during sensor deployment, post-sensor deployment) in each community to provide training, discuss data, and gather feedback to inform the development of the Sensor Educational Toolkit. The first workshop, the “introduction to the project” workshop, included an introduction to the project and air quality sensor technology. The second workshop, the “pre-sensor deployment” workshop, included training on sensor installation and the distribution of sensors. The third workshop, the “during sensor deployment” workshop, included data analysis, discussions of results, and an opportunity for participants to provide feedback. The fourth workshop, the “post-sensor deployment” workshop, included a discussion of outcomes and lessons learned. Each workshop was 1.5–2 h long, and they were held in person unless this was not possible due to an external factor or unexpected circumstance. In nearly all communities, the workshops were conducted as described above. An exception is communities B, C, and F, where community group leads requested that the first and second workshops be combined into a single meeting. Additionally, in some communities, the third and fourth workshops were conducted remotely or were combined. In all cases, this change in meeting format was either at the request of community group leads or in response to restrictions on in-person gatherings during the COVID-19 pandemic. 

Project leads at the South Coast AQMD led all workshops, except for those occurring in the two northern California communities K and M, where the Bay Area AQMD led interactions and workshops. Translation at meetings was provided as requested by community groups. Many of the project materials were made available in Spanish. In northern California, languages requested by community members included Cantonese, Filipino, Khmer, Korean, Mandarin, and Vietnamese, necessitating further translation of project materials. 

All participants in all communities were provided with the same background information, materials (sensors, resources such as installation guides), training, and series of workshops; however, additional in-person and over-the-phone support (ranging from assistance with troubleshooting to replacing sensors to tutorials on data analysis tools) was provided as requested by community partners. Throughout the project, leads at the South Coast AQMD made every effort to identify and meet the needs of each individual community. As sensors were distributed on a one-to-one basis, in most cases, sensors were deployed by community members. The deployments in communities C and L are the exception, where sensors were deployed by project staff from the South Coast AQMD in coordination with the community. Project leads at the South Coast AQMD assessed rates of participation and engagement by tracking sensor installation rates and by examining the quantity and continuity of the sensor data. Feedback was gathered directly from community members through surveys and discussions. One set of surveys was developed using ESRI Survey123 (source: https://www.esri.com/en-us/arcgis/products/arcgis-survey123/overview, accessed on 15 February 2022) and distributed electronically to those who agreed to participate in the project. These included an installation survey, a non-installation survey, and a logbook survey. The installation survey recorded information about when, where, and how the host installed the sensor. Survey123 allowed for the collection of geospatial information (latitude and longitude) along with pictures of the sensors installed, which is helpful to remotely review whether siting may be a concern, especially when sensors are not following typical community trends. The non-installation survey sent out following the installation survey was intended to provide participants, who had not installed their sensor, an opportunity to share their reason and request assistance if they wished. The logbook survey was intended for ongoing use and gave participants a tool to record air quality events and observations, which could aid sensor data analysis and interpretation of the results. The project leads did not request that participants complete a certain number of logbook surveys; it was offered as a supplemental resource. 

Discussions were conducted and in-person surveys were collected during the workshops (offering multiple formats for participants to provide feedback to the project leads). Some communities also provided additional information in progress reports and summaries. All reports and detailed summaries of each workshop were reviewed, and responses and comments from the communities were categorized according to relevant project phase and are summarized in the results below. The design of questions for the electronic and in-person surveys/discussions was guided by the project’s objective of determining how to provide communities with the necessary knowledge to appropriately select, use, and maintain sensors and correctly interpret the data collected by sensor networks.

## 3. Results and Discussion 

This section provides an overview of the results, organized by project phase, and highlights those which may be valuable for future air quality sensor projects. Furthermore, the methodology and results of the project informed the development of the Sensor Educational Toolkit, which includes CCS resources for all project phases necessary to developing a community air monitoring network. The Sensor Educational Toolkit includes a Guidebook, “Community in Action: A Comprehensive Guidebook on Air Quality Sensors”, three training videos, data analysis and visualization tools, and a number of resources and documents developed during the STAR Grant project (e.g., workshop slides, surveys and forms, installation guides, infographics, reports created by communities, etc.). All of these tools and resources are available to be used as is or adapted by future CCS air monitoring projects (http://www.aqmd.gov/aq-spec/special-projects/star-grant, accessed on 15 February 2022).

### 3.1. Planning and Preparing for a Project

**Figure 1:**
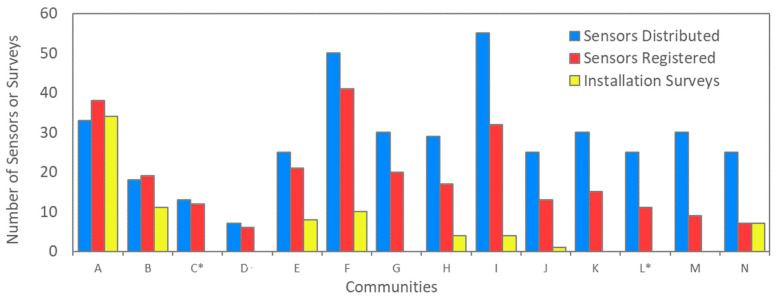
Number of sensors distributed (in blue) and installed (in red), and number of installation surveys submitted (in yellow); the asterisk indicates that a different model was used for the deployment where the installations were not undertaken by community members.

**Figure 2:**
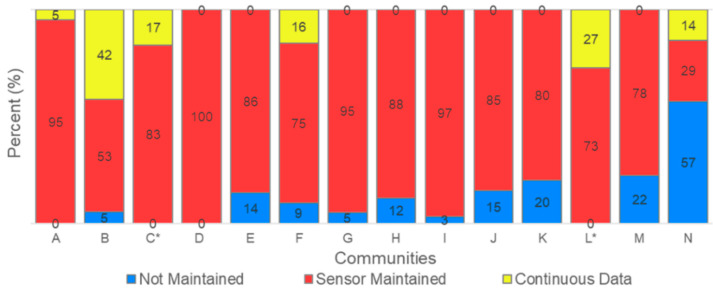
Sensor maintenance statistics, listed by community; the asterisk indicates that a different model was used for the deployment and installations were not undertaken by community members.

The rates of participation and engagement varied across the communities. This variation is illustrated in Figure 1, which compares the number of sensors initially distributed (in blue) to the number installed (in red). Figure 1 also includes the number of installation surveys completed by each community. Communities are ordered by installation rate, from highest to lowest. The installation rate ranges from approximately 30% to 115% (in a community where additional sensors were requested). For context, the original goal was to engage 25–30 project participants in each community. The average time to install sensors also varied among the communities, ranging from 21 days to 416 days (Table S1). Only seven responses to the non-installation survey were received; thus, it did not provide significant insights into the variability in installation rates between communities. Figure 2 illustrates the levels of engagement after installation based on the amount of data collected by each sensor. Participants were trained on using the real-time data portal and were advised to use this tool to monitor air quality conditions and whether the sensor was reporting data. For each community, the percentage of sensors that ran continuously (yellow), were maintained (red), or were not maintained (blue) are shown. Sensors that were in operation for at least 6 months without interruption are considered to have collected “continuous data”. Sensors in operation for at least 10 days (often far longer) that experienced an interruption to the data logging but were brought back online at least once are considered “maintained”. Lastly, sensors that operated for less than 6 months and were not brought back online when they stopped logging data are considered “not maintained”. Disruptions that last 30 min or more are typically caused by issues related to Wi-Fi or power connections that require user intervention. However, it is possible that, in some cases, the sensors reconnected automatically.

In general, greater effort to maintain sensors seems to be associated with high installation rates. However, it is noteworthy that, once the sensors were installed in a community, most participants were willing to maintain their sensors. The sensor used for this project does not notify the user if data logging is interrupted; thus, participants were likely using the data portal to notice these interruptions. 

**Figure 3:**
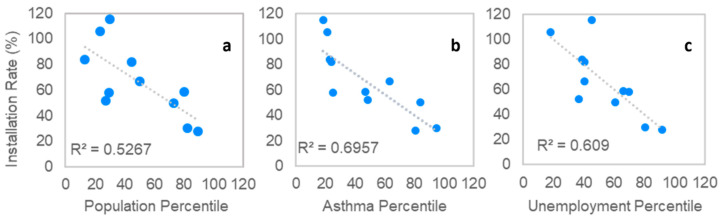
Relationship between (**a**) overall CalEnviroScreen population characteristics percentile, (**b**) asthma rates, (**c**) unemployment rates and installation rates in the communities. Note that all communities are included with the exception of those three indicated in Figure 4 and Figure 5 as sensors were not installed by community members.

To better understand these trends, we considered the environmental and socioeconomic factors impacting those communities using data from CalEnviroScreen 3.0 (California Office of Environmental Health Hazard Assessment, 2020). The three factors which displayed the strongest relationships to installation rates (as indicated by the *R*^2^ values) were the overall CalEnviroScreen population characteristics percentile values, asthma percentile values, and unemployment percentile values (Figure 3a–c). In other words, higher proportions of asthma, unemployment, or a higher overall socioeconomic burden appears to be associated with lower installation rates. Plots examining all other metrics are available in the Supplementary Materials (Figures S4–S14). 

Given that the same materials, training, and support were made available to all communities, the difference in participation may highlight how communities facing higher burdens could benefit from additional support. Facilitating access to these tools is especially important as these types of communities can face environmental justice issues such as disproportionate exposure to pollutants and may stand to benefit substantially from the successful use of this new technology. As observed by Harrison et al., strong organizational capacity is key to the success of participatory air monitoring projects involving complex, conventional air monitoring tools [35]. Thus, research partners should carefully assess community needs, resources, and capacity before a project. Future air sensor projects may also benefit by looking to established participatory research methods to learn about project structures and building partnerships [30,50]. A few specific considerations shared by community group leads include translation needs and barriers to recruitment noted in Section 3.2.

A notable difference from past research is the departure from conventional monitoring methods. Air quality sensors are consumer products, and, while sensors are readily available to the public, as shown here, they are not necessarily easy to integrate into community-based work for all populations. Until these technologies become more accessible (i.e., easier to use), future projects should especially focus on supporting and facilitating the initial installation of devices (e.g., first choosing the most appropriate sensors, then the physical setup of the devices). 

**Figure 4:**
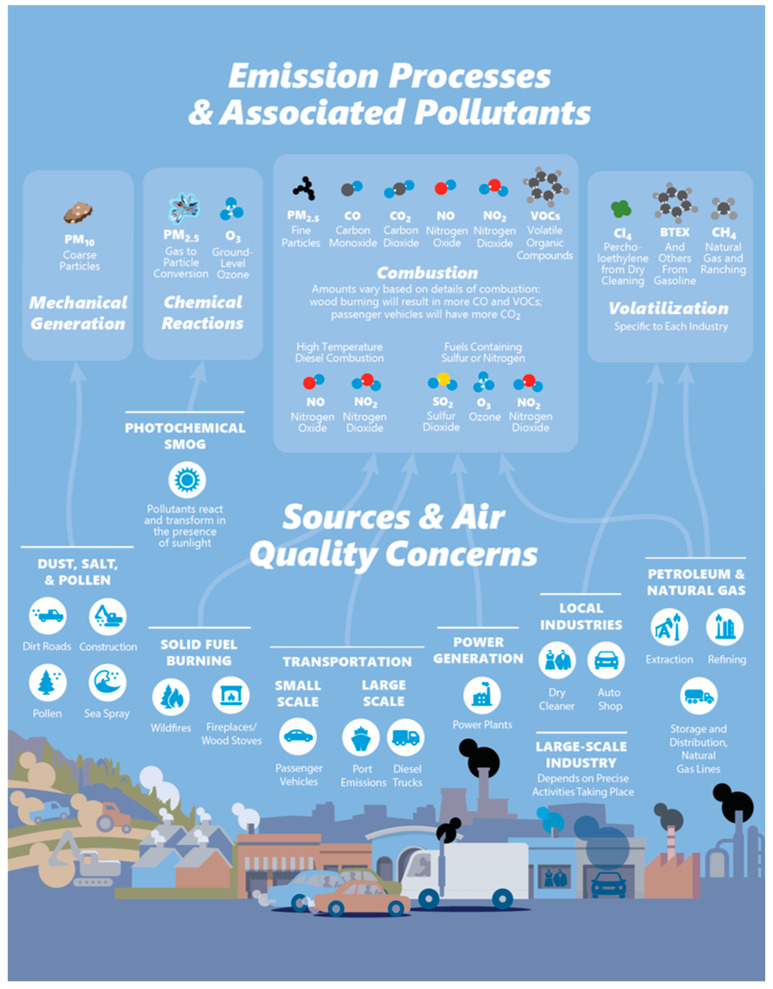
One-page resource to help communities identify pollutants of concern, from the guidebook “Community in Action: A Comprehensive Guidebook on Air Quality Sensors”.

**Figure 5:**
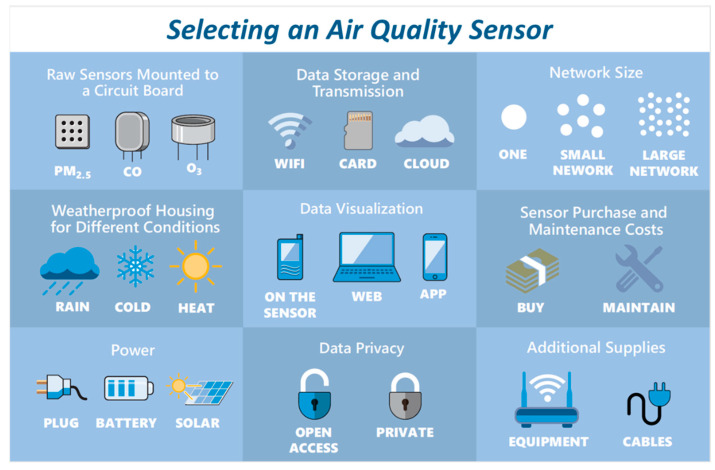
Graphic on considerations for sensor selection, from the guidebook “Community in Action: A Comprehensive Guidebook on Air Quality Sensors”.

These experiences informed an in-depth discussion on planning in the Guidebook of the Educational Toolkit and the creation of several resources. The discussion includes topics such as considering different approaches to project organization, learning more about your community’s air quality concerns (i.e., what pollutants to target), evaluating your resources, and selecting a sensor or sensors to meet project needs (this information is in Chapters 2 and 3 of the guidebook). The information is communicated using a combination of text and visuals to ensure it is accessible, with references to external sources where appropriate. The guidebook also offers useful one-page resources and graphics that can be used as excerpts (e.g., Figure 4 and Figure 5). Two of the training videos may also be valuable at this stage as they introduce air quality background information and monitoring, as well as the use of sensors in CCS projects. These resources in the Sensor Educational Toolkit will help communities and their research partners consider all aspects of a sensor project from the outset, setting them up for success.

### 3.2. Deploying and Maintaining Sensors 

Notably, all communities reported similar technical difficulties, including those with high and low rates of participation, and those of different demographic and socioeconomic composition. Individuals in all communities remarked on the technical difficulty of completing the steps necessary to set up and install their sensors. In many cases, sensor hosts required assistance to complete installations. Communities with higher installation rates (A, B, E, F, G, and I) shared that a single community lead, community group staff, or a community member with technical expertise installed all or most of the community’s sensors. Thus, the higher rates of installation illustrate the value of using a model where local experts and experience are leveraged. Further highlighting the value of strong community leadership, an unexpected safety issue required a batch of power cords to be recalled and replaced in 8 of the 14 participating communities. This required community group leads to distribute replacement cords and assist with bringing the sensors back online. Another issue related to installation was the mislabeling of sensors. If a sensor was not registered using the expected identification label, it could not be tracked using the open-access data portal and, consequently, community group leads were not able to view or access the data. During sensor selection, project leads should consider whether this issue can occur with the selected device and draft solutions.

**Figure 6:**
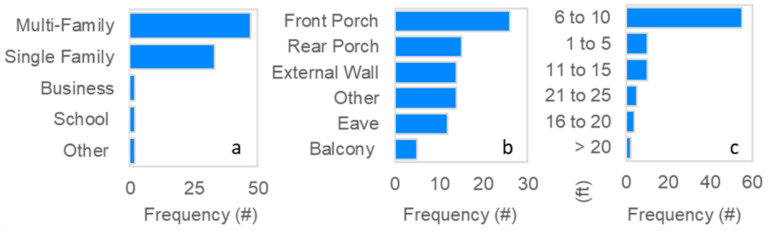
Details of installed sensor locations: (**a**) type of building, (**b**) location at building, and (**c**) height from ground in ft (*n* = 86, installation survey responses).

To collect more feedback on sensor installations, a survey was deployed. Participants returned a total of 86 electronic installation surveys (Figure 1). This survey shed light on where and how participants installed sensors; sensors were primarily installed at residences, 6–10 ft off the ground (Figure 6).

Participants were also invited to submit a photograph of the installed sensors, and 28 were received (Figure S15). The photos were assessed to determine whether siting and installation recommendations were followed. In general, sensors were installed appropriately. Typical recommendations include installing sensors where they have access to free airflow, at least 3–6 ft off the ground, and away from building surfaces (Williams, et al., 2014). Our team also instructed participants to install sensors away from any sources of particulates on their property that might influence the data. According to the photos submitted, no sensors were installed close to the ground, with 16 out of 28 photos showing a sensor installed with access to representative airflow. Some were installed against a surface in a sheltered location (e.g., on a mostly enclosed porch). One sensor was installed close to a potential confounding source (a large air conditioning unit; see Figure S15d), and another nine sensors were installed in areas free of possible influences. The remaining photos did not include the surrounding area.

Participants noted poorly sited external power outlets, a lack of external power outlets, and the variable strength of the Wi-Fi signals outside their homes as limiting factors. Related to this feedback, community group leads also noted that it was difficult to recruit renters, as renters and lower-income residents may not have Wi-Fi at home or an outdoor power source, and some rental agreements do not allow for installing a sensor. Given the logistical challenges of installing a sensor at a home, it may be beneficial for future research to assess the impact of installation in a non-ideal location. In addition, we recommend that future projects establish an iterative siting process, so that participants can adjust siting on the basis of feedback from project leads. Sensor selection may also impact installation rates in that sensors with fewer requirements would likely have higher installation rates. For example, a solar-powered and cellular-connected sensor would remove some of the limiting factors described here (i.e., availability of external power and Wi-Fi signal strength), but may also result in a higher initial cost per sensor. Furthermore, utilizing participatory design and the study of HCI could also result in more easy-to-use and easy-to-install sensors [24,27].

During the deployment, nearly all communities reported that some sensors had problems maintaining connectivity in terms of Wi-Fi, power, or both. In addition to logistical issues, additional feedback was provided by communities in workshop discussions and reports. Leads from six communities expressed that the effort needed to provide technical support and maintain the project’s momentum was greater than anticipated. Ensuring adequate funding for community groups will help these projects to be successful, which can result in higher data recovery and data quality. According to feedback from community group leads, funding allocation should consider support for staff time, as well as equipment such as replacement sensors or other accessories (e.g., extension cords, power splitters, Wi-Fi range extenders). To maximize engagement and data recovery, community participants also recommended that future projects build notification systems to alert users when a sensor has gone offline or when potential air quality events occur. The notification could also include ways to troubleshoot and restore connectivity. A few participants also requested instructions for cleaning the sensors to either troubleshoot apparent malfunctions or as part of regular maintenance. During the project, this information was provided as requested, but future projects may wish to develop this documentation ahead of time. Lastly, the following were identified as ways in which project leads can assist community partners: identifying mislabeled project sensors, helping troubleshoot technical issues with sensors, and providing access to supplemental data (i.e., air quality or meteorological data from nearby air monitoring stations).

**Figure 7:**
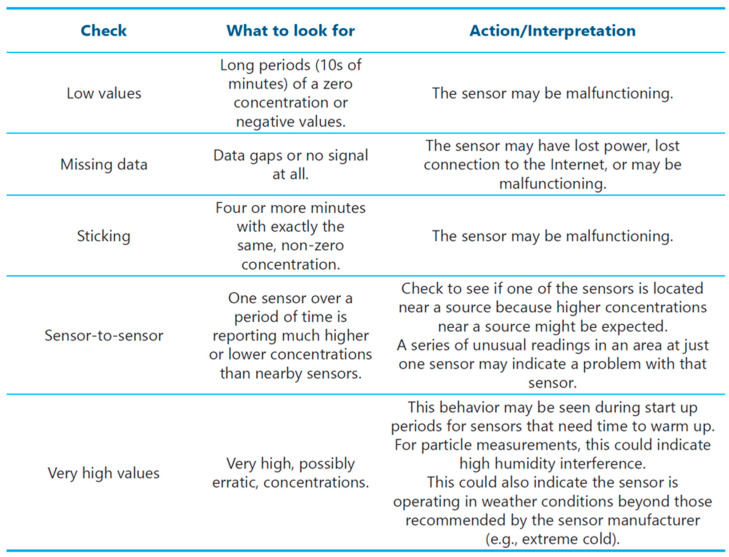
List of checks to assist with ensuring high data quality, from the guidebook “Community in Action: A Comprehensive Guidebook on Air Quality Sensors”.

In response to these lessons, a number of resources available in the educational toolkit were developed to support sensor use. The Installation Guide (available in English and Spanish) was revised iteratively throughout the project shaped by feedback from community members, and the final versions are available in the guidebook appendix. This guide is ready for studies using the same sensor device, or it could be used as a model for developing installation guides for other types of sensors. In addition, there is a training video available demonstrating sensor installation and sharing general advice regarding siting. Furthermore, copies of all surveys used are available to be adapted by future projects. In Chapter 4, the guidebook advises on a range of considerations related to installation and maintenance, including all those concerns discussed here and others such as data quality. Advising on tracking data quality, the table in Figure 7 (from Chapter 4 of the guidebook) includes a generalized list of recommended checks compatible with any sensor device; also included is a list of sensor “state-of-health” metrics customized for the specific sensor used. Together, these resources can help projects develop comprehensive plans that help ensure the collection of high-quality, useful data. 

### 3.3. Data Access, Analysis, and Communication

**Figure 8:**
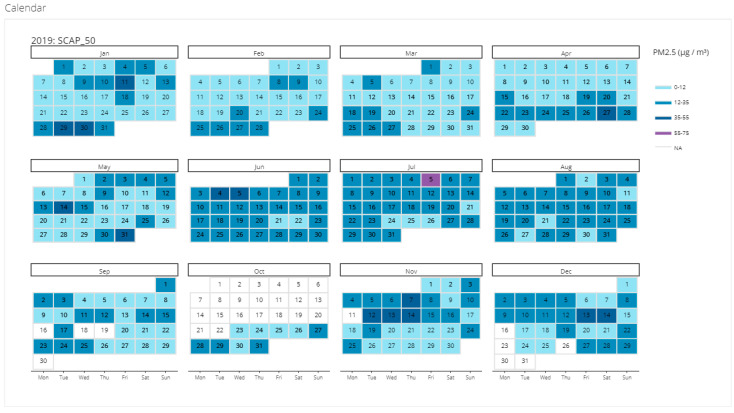
Example of a calendar plot, the most effective data visualization identified during the project.

In terms of communicating results, project leads used a variety of formats to share the data in workshops, including presentations and printed infographics that piloted different data visualizations (Figure S16). Community members consistently displayed the highest engagement with calendar plots (Figure 8), with these plots generating lively conversation among workshop attendees. Future projects should consider using this visual for nontechnical audiences to summarize data. This engagement was likely due to the intuitive nature of these plots; in other words, it was easy for participants to make connections between the data and their own past experiences or events. 

Other feedback from discussions included comments on the difficulty of accessing the historical sensor data, which, for this sensor, requires technical skills. Participants from several communities reported that their interest decreased over time due to the effort required to access and view the data using the data portal. Similarly, in workshop surveys, respondents were asked how often they check their sensor data and if they had questions about what the data meant, and the most common answer to both was “sometimes” (Figure S17). Community group leads expressed a desire for the capability to create easy-to-interpret plots and statistics to share with the community. For example, an auto-generated pdf or report that could be sent out biweekly to provide a quick summary of recent data would meet this request, or a platform that allows leads to generate and save their own plots. 

**Figure 9:**
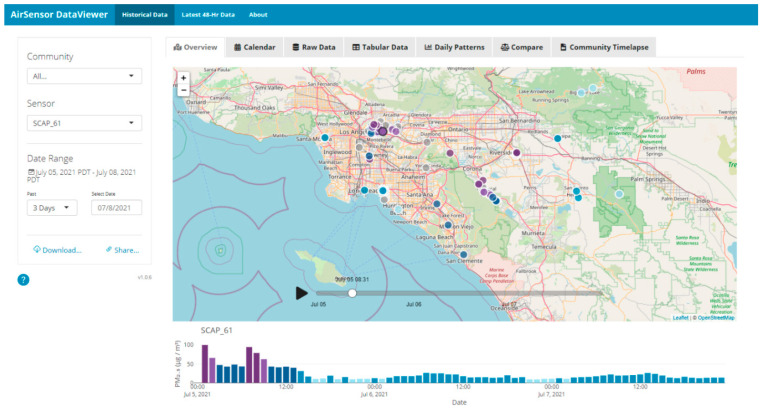
Screenshot of the DataViewer tool (version 1.0.1) built for community members to explore current and historical data.

This feedback highlighted an opportunity to expand and enhance users’ ability to engage with and interpret sensor data. In response, the open-source AirSensor package (version 1.0) for the R programming language and a web-based DataViewer application (version 1.0.1) were developed to facilitate data access, analysis, and visualization [32,51]. The AirSensor package includes functions that provide easy and direct access to both current and historical sensor data and functions to process data in a standardized way (e.g., QA/QC algorithms), which enables advanced data analysis. The DataViewer tool, built using the AirSensor package, is an intuitive user-friendly web-based solution that offers an engaging interface and visualizations, as well as access to current and historical data (Figure 9 provides a screenshot of the interface). These solutions increase the usefulness of the sensors by increasing the accessibility and interpretability of the data, especially the historical data [32].

Another important observation from the survey responses was that most participants stated that they never looked at other sources of air quality information (Figure S17). Thus, future projects may consider familiarizing community members with existing air quality resources that provide health-based information (e.g., AirNow, https://www.airnow.gov/, accessed on 15 February 2022). This information can complement the information offered by the locally deployed sensors, although education and context explaining the difference between technologies and data processing will need to accompany these resources. As the interest in hyper-local air quality information by communities grows, we are likely to see regulatory agencies incorporating sensor data into their data displays. One example is a real-time AQI map that blends regulatory data, sensor data, and models to provide AQI values at a 5 km × 5 km resolution [52]. Another example is the Fire and Smoke Map developed by the U.S. EPA [53]. 

In addition to the AirSensor package and the DataViewer tool, in Chapter 4, the guidebook includes an introduction to data analysis and different plot types, as well as an overview of publicly available tools to support data engagement. The guidebook also contains an example analysis for a specific air quality event, modeling an approach to exploring and interpreting air quality data. Other resources include a sensor-agnostic data analysis guide, a user guide for the DataViewer, and an example infographic. It is critical for projects to consider the ease and method of data access and visualization in relation to project goals (e.g., environmental education and outreach or the collection of high-data quality), as these factors can significantly influence overall project success.

### 3.4. On the Usefulness and Value of Sensors 

**Figure 10:**
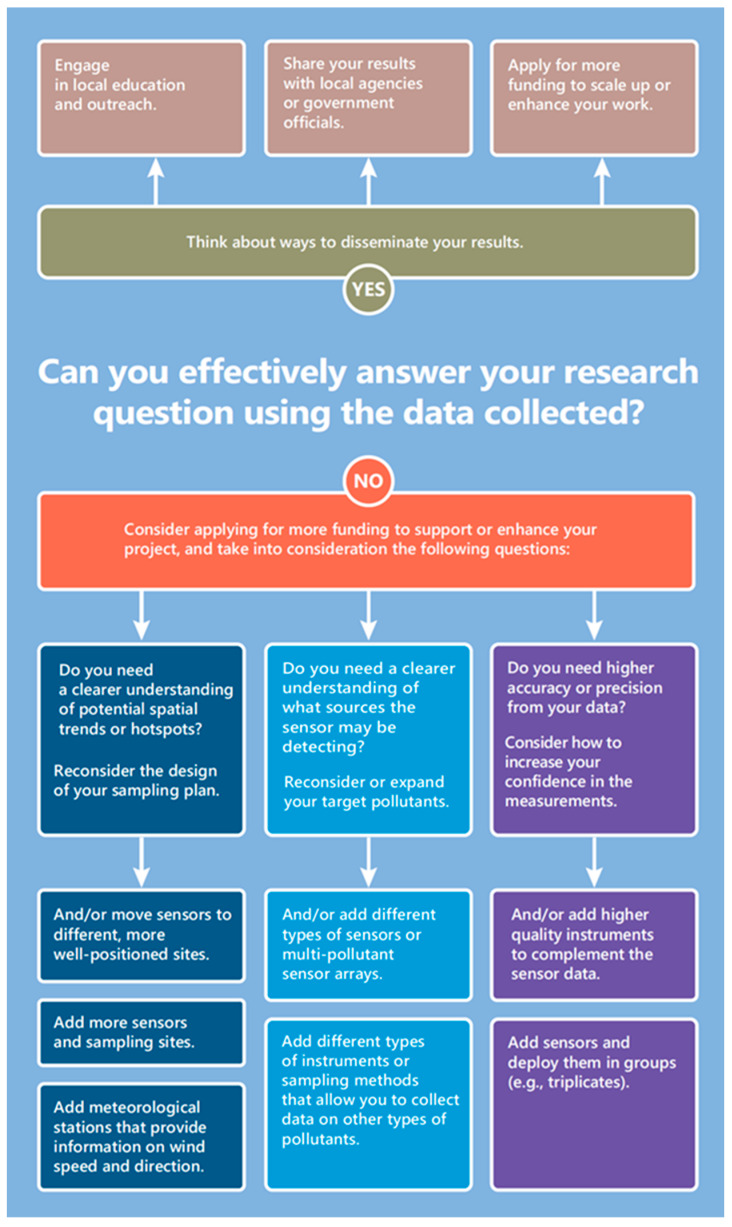
A decision tree to help projects consider possible next steps, from the guidebook “Community in Action: A Comprehensive Guidebook on Air Quality Sensors”.

Once the sensors were in use, most respondents (90%) found their sensor to be “somewhat” to “very” useful. When asked if respondents noticed a relationship between the data and activities (e.g., nearby grilling, or a wildfire), the majority responded “sometimes” or “often”, affirming that sensors have the potential to increase users’ understanding of their local environment (Figure S17). Prior to participating, most respondents expected that they would change their behavior (e.g., by reducing their contributions or exposure to emissions) on the basis of air quality data (76%); however, a much smaller portion reported having done so (32%) (Figure S18). These results suggest a lack of understanding around how to translate the data into actions that may improve air quality. In response, Chapter 5 of the guidebook presents activities to consider upon the completion of a project. These include a list of ways to take action to reduce emissions and exposure (e.g., active programs that could serve as models), advice for communicating results to different audiences (e.g., local regulatory agencies), and guidance regarding next steps (e.g., Figure 10 depicts a decision tree from Chapter 5). Given that air quality sensors are often used for pilot projects or in networks that grow over time, it can be useful to assess whether the data from the deployed network is contributing to the initial research goals. Tools such as the “Handbook for Citizen Science Quality Assurance and Documentation” developed by the U.S. EPA [54] can help members of the public work through the process of defining these goals in detail. For example, this document includes guidance on defining data quality objectives and data quality indicators, which can help sensor users assess the adequacy of their data. If users decide the collection of additional data is necessary, it may be helpful to review other sensors studies. For example, a study where sensors were placed around an airport illustrates the importance of careful siting, as well as how to incorporate supplemental data such as wind speed and direction [55]. This same study also illustrates how the data from different gas-phase sensors can be leveraged to better understand sources, in this case using source apportionment [55]. In AQ-SPEC field and laboratory evaluations, sensors are always tested in triplicate. The analysis associated with these evaluations can illustrate ways to assess the variability within these triplicates to in turn better understand sensor accuracy and precision [6,7]. The guidebook both points users to the “Handbook for Citizen Science Quality Assurance and Documentation” as a useful resource and includes an in-depth discussion of these different potential next steps. 

In addition to changing behavior, community members asked how these data could be used to seek stronger environmental regulations, which led to a discussion of the limitations of air quality sensors and current suitable applications. During the workshops, participants shared anecdotes that highlighted how sensors can provide useful information despite current limitations. Multiple participants from different communities described using the sensor data to decide whether to exercise indoors or outdoors and when to walk their dog, commenting on the value of access to locally relevant data. One community described using the sensors to monitor controlled burns and notify nearby schools of emissions, noting the utility of real-time data. Another community using paired indoor and outdoor sensors discussed using the data to adjust cooking behaviors and optimize the use of indoor filtration units [56]. These reported uses are similar to those seen in another project using particulate matter sensors [43], suggesting that developing materials on the basis of these examples could help participants in future projects make more effective use of sensor data, resulting in reduced exposure and emissions.

The inclusion of experiences and perspectives of 14 different communities, including first-hand examples of community-led analysis, products, and strategies, makes this educational toolkit unique. These examples provide a few case studies in which community members took initiative to analyze data and derive information from the sensor network, highlighting the value of open access data. One community member assessed the performance of a sensor under local conditions and developed a correction algorithm to increase the accuracy of the community’s data. This work also helped identify an important limitation of the sensor; it was seen to underpredict pollutant levels in high-wind conditions associated with dust events (Figure S19). In another community, a participant with technical and programming skills created an animation of the sensor data (Figure S20) that depicted emissions of a nearby wildfire, which was shared among the community to help those with less technical backgrounds better understand the impact of smoke in their community. A student in another community used the sensor data for a science fair project and presented at one of the workshops. Seeing the data presented by a student from their own community enhanced the attendees’ connection to and interest in the information. These examples also highlight an important principle of community-based participatory research—identifying existing skills, assets, and knowledge within a community at the outset of a project [30]. Leveraging different skills and abilities, whether technical or organizational, can strengthen projects and increase their potential for lasting positive impacts. Thus, enhancing existing capacity and building new capacity in the participating community should be a key consideration for future sensor projects.

### 3.5. Additional Strategies for Successful Projects

Lastly, we share several strategies that communities used to enhance their work on the STAR Grant project that may serve as models for future projects. Community F successfully applied for additional funding to expand their sensor network and continue monitoring local air quality after the conclusion of the STAR Grant deployment. This same community also established a partnership with a program for undergraduate students at a nearby college. These students analyzed data and developed a report for the community that directly addressed their questions. This partnership was mutually beneficial, as the students gained experience with a real-world dataset and developed data science skills. 

**Figure 11:**
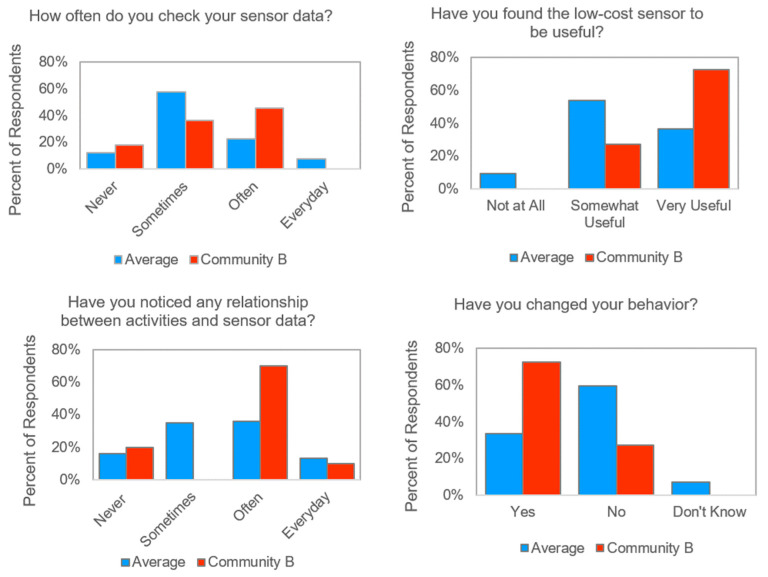
Comparing workshop survey responses for the average (in blue, *n* = 63) to Community B (in red, *n* = 11); specific questions are listed at the top of each chart. Note that Community B was the only community in which paired indoor and outdoor sensors were used.

Several communities also found value in multiple sensors or additional monitoring. In Community B, a higher proportion of participants reported checking their data “often”, noting a relationship between activities and the sensor data “often”, finding their sensors to be “very useful”, and changing their behavior, (Figure 11). In discussions, there was agreement that this was likely due to the presence of paired indoor and outdoor sensors, which allowed participants to better understand how their individual actions relate to air quality. 

Multiple sensor types or supplemental monitoring can provide greater insight into specific sources. In one community, partners conducted supplemental monitoring using research-grade instruments to understand potential impacts of a nearby freeway. This supplemental monitoring was undertaken to address a limitation of the sensor used, which is not well suited to efficiently detect ultrafine particles typically associated with vehicle emissions—a concern for the community. Early in the project, another community expressed difficulty using the provided low-cost PM sensors and an interest in gas-phase pollutants. The project leads provided this community with multi-sensor units, the Aeroqual AQY v0.5. The sensor installation was undertaken jointly by a community leader and Aeroqual staff to ensure appropriate installation and operation of the devices. The addition of these multi-sensor units provided the community with more data to make sense of local air quality issues. These multi-sensor units were also added in several other communities. Other studies have also observed the value of multiple sensors and multi-sensor systems [26,57], affirming that this may also be a useful strategy for future projects.

## 4. Conclusions

The communities participating in this project demonstrated a willingness to engage with a new air monitoring technology. Their participation helped to identify specific challenges to using sensors and to develop strategies that lead to success. Although commercial air quality sensors are intended for use by the public, as demonstrated here, additional support is needed to ensure proper sensor siting, installation, and ongoing sensor maintenance. Furthermore, projects must consider data quality, data access (real-time and historical) and the suitability of data, visualization, and communication solutions for their community partners. Until the usability of sensors improves, we have attempted to address identified needs through our Sensor Educational Toolkit. We anticipate that this toolkit will be broadly applicable as its development was informed by a large-scale, multi-community project involving a diverse range of participants. The key component of the toolkit is the guidebook, which includes practical guidance for every stage of an air quality sensor project—from project planning to taking action informed by the results. This guidebook is supported by a range of other resources, all available on the AQ-SPEC website (http://www.aqmd.gov/aq-spec/special-projects/star-grant, accessed on 15 February 2022). The educational toolkit is available to support community and citizen scientists in designing sensor networks to collect high-quality, useful, and locally relevant data. 

While continued research into sensors, sensor performance, and sensor network applications is needed, the results presented here provide current perspectives and specific examples of successes and limitations in the implementation of air quality sensor networks for community-based monitoring. These perspectives and examples have the potential to catalyze improvements in the design of sensors and assist in the development of CCS sensor networks. Given the continual advancements in next-generation environmental monitoring technologies, this work may also inform further research into how to increase the accessibility and impact of this technology from the perspective of a citizen scientist.

## Data Availability

Deidentified and aggregate data are available in the article and supplementary materials; please contact the corresponding author with any additional questions regarding data access and availability.

## Supplementary Materials

The following supporting information can be downloaded at https://www.mdpi.com/article/10.3390/s22072543/s1: Figure S1: Reponses to question: “What sources are you concerned about?” (*n* = 86); Figure S2: Excerpt from the PurpleAir PA-II sensor field evaluation report, illustrating agreement between sensors and a co-located reference instrument, full report available on the AQ-SPEC website [58]; Figure S3: Average population percentiles for each participating community, from CalEnviroScreen, a higher percentile indicating a greater burden for that index in that community and illustrate the diversity among participating communities; Figure S4: Overall Pollution Percentile Values; Figure S5: Ozone Percentile Values; Figure S6: PM_2.5_ Percentile Values; Figure S7: Diesel Percentile Values; Figure S8: Overall Population Percentile Values; Figure S9: Asthma Percentile Values; Figure S10: Poverty Percentile Values; Figure S11: Unemployment Percentile Values; Figure S12: Education Percentile Values; Figure S13: Linguistic Isolation Percentile Values; Figure S14: Overall CalEnviroScreen Score; (The Figures S4–S14 depict CalEnviroScreen 3.0 statistics for each community with respect to the installation rates for that same community [59]. Note, all communities are included except for those two indicated in Figure 1 and Figure 2, where sensors were not installed by community members). Figure S15: Here the range of installations by participants are shown. In (a,b) the sensors are elevated and have access to free airflow (though the participants would need to note vehicles entering and exiting the garage in the photos on (b)); these are examples of proper installation. In (c) the sensor is elevated but is somewhat sheltered and access to airflow is limited. In (d) the sensor is above an air conditioning unit that may produce dust and serve as a confounding source, this would be an example of improper siting. While participants from community. A submitted most surveys (34 surveys), 45 surveys were submitted by participants in other communities; Figure S16: Example of an infographic designed for and shared with one of the participating communities; Figure S17: Results from during-the-deployment survey, for the question: (a) “Do you look at other sources of air quality information?”, (b) “Do you regularly look at the low-cost air quality sensor data?”, (c) “Do you have questions about what the data mean?”, and (d) “Have you noticed any relationship between activities and sensor data?” (*n* = 63); Figure S18: Results from the prior-to-deployment survey, for the question, (a) “Do you think you would change your behavior based on air quality sensor data?”, (*n* = 50) and results from the during-the deployment survey, for the question, (b) “Have you changed your behavior?” (*n* = 63); Figure S19: Examples of data analysis examining sensor performance and the influence of wind speed, conducted by a community member; Figure S20: Screenshot of a data animation showing a wildfire event, the height of the red bars indicated pollutant concentrations, while the size of the blue circle indicates wind speed; Figure S21: number of surveys completed prior-to-deployment (in blue) and during-the-deployment (in red) in each community, **denotes communities where either workshops were not held due to the use of a different sensor deployment model or surveys not being distributed/collected by team leading the workshop; Figure S22: Aggregate ages of the survey respondents for the prior-to-deployment survey (in blue) and during-the-deployment survey (in red); Figure S23: Aggregate ethnicity of the survey respondents for the prior-to-deployment survey (in blue) and during-the-deployment survey (in red); Figure S24: Aggregate education level of the survey respondents for the prior-to-deployment survey (in blue) and during-the-deployment survey (in red); Figure S25: Number of people living in the home for the prior-to-deployment surveys (in blue) and during the-deployment surveys (in red); Table S1: Average Time to Install Sensors (by Community).
