# Supplementary material for: Towards the Development of a Sensor Educational Toolkit to Support Community and Citizen Science
Source: Sensors (Basel). 2022 Mar 26;22(7):2543. doi: 10.3390/s22072543 (PMC9003123; doi:10.3390/s22072543)
Supplement: Supplementary file 1 [file sensors-22-02543-s001.zip › sensors-1624806-supplementary.pdf]

Supplementary Materials: Towards the Development of a Sensor Educational Toolkit to Support Community and Citizen Science

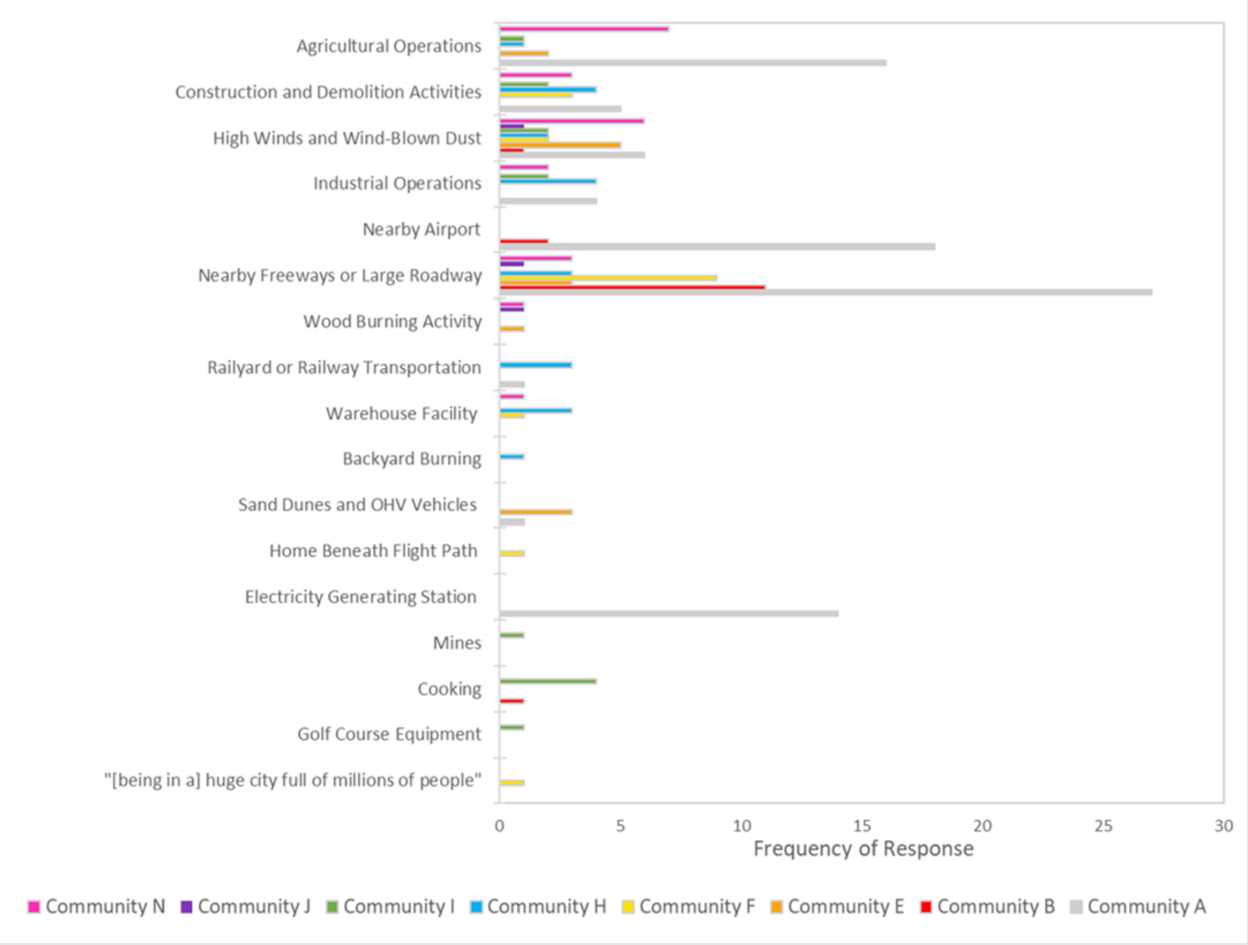

Figure S1: Reponses to question: “What sources are you concerned about?” (n = 86)

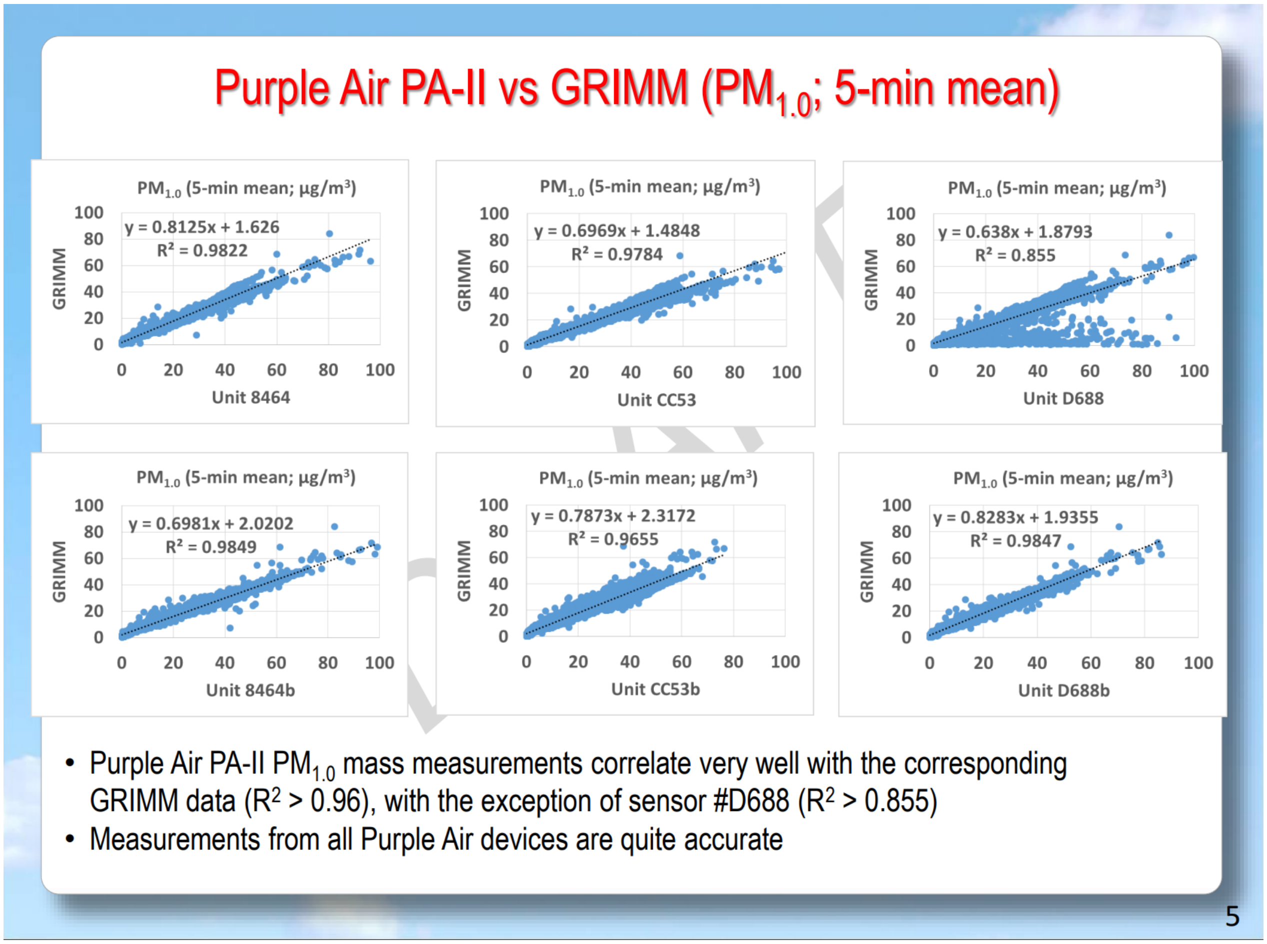

Figure S2: Excerpt from the PurpleAir PA-II sensor field evaluation report, illustrating agreement between sensors and a co-located reference instrument, full report available on the AQ-SPEC website [58].

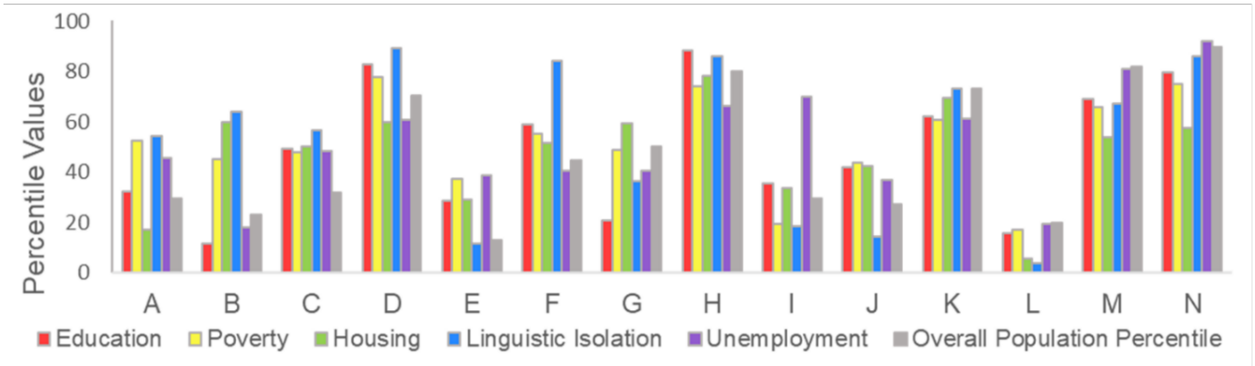

Figure S3: Average population percentiles for each participating community, from CalEnviroScreen, a higher percentile indicating a greater burden for that index in that community and illustrate the diversity among participating communities

Calculation of Average Percentile Values: For each community, the percentile values for census tracts where a sensor was sited are averaged to calculate an estimated value for the community. The data shown is from CalEnviroScreen 3.0, and the percentile values indicate how a community compares to the rest of the state of California, with a higher percentile indicating a greater burden for that index in that community [59].

Table S1 – Average Time to Install Sensors (by Community)

Community	Distribution Date (mm/dd/yyyy)	Average Installation Date (mm/dd/yyyy)	Number of Days
A	10/09/2017	12/06/2017	58
B	10/25/2017	12/15/2017	51
C*	N/A	N/A	N/A
D*	N/A	N/A	N/A
E	04/19/2018	06/19/2018	61
F	01/19/2018	04/22/2018	93
G	01/10/2018	02/09/2018	30
H	03/28/2018	04/27/2018	30
I	07/26/2018	08/23/2018	28
J	04/19/2018	05/10/2018	21
K	12/19/2017	02/08/2019	416
L*	N/A	N/A	NA
M	12/19/2017	12/07/2018	353
N	12/12/2017	05/28/2018	167
		**Average**	**119**

Note, dates of a sensor replacement were not included, thus the average dates are based on the first-time installation of all network sensors only. *(indicates a different model for deployment was used and sensors were not installed by community members)

Installation Rates vs. CalEnviroScreen Data

The following figures (S4 – S14) depict CalEnviroScreen 3.0 statistics for each community with respect to the installation rates for that same community [59]. Note, all communities are included except for those two indicated in Figures 1 and 2, where sensors were not installed by community members.

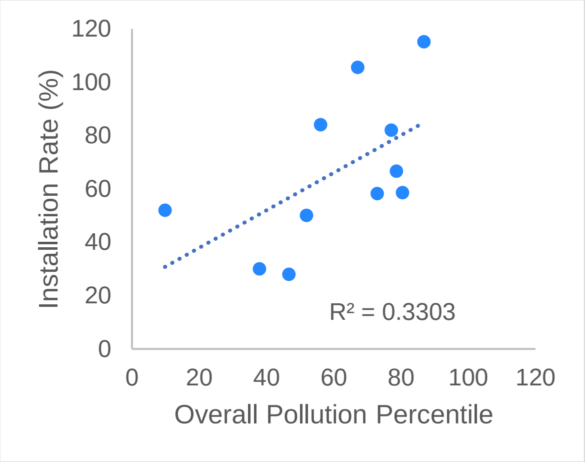

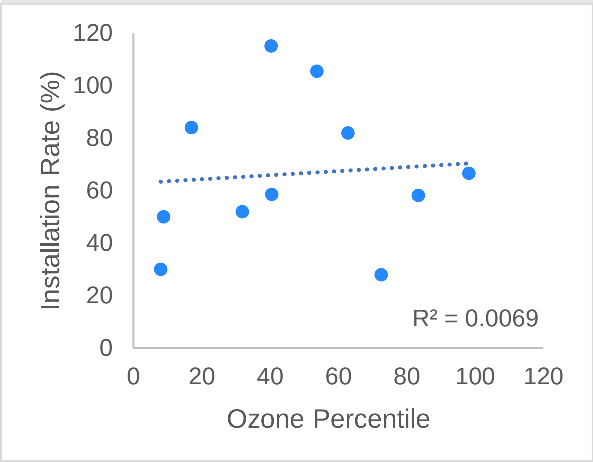

Figure S4 (left) – Overall Pollution Percentile Values; Figure S5 (right) – Ozone Percentile Values

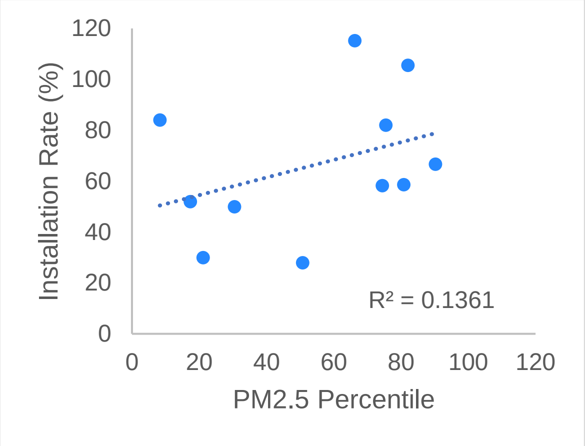

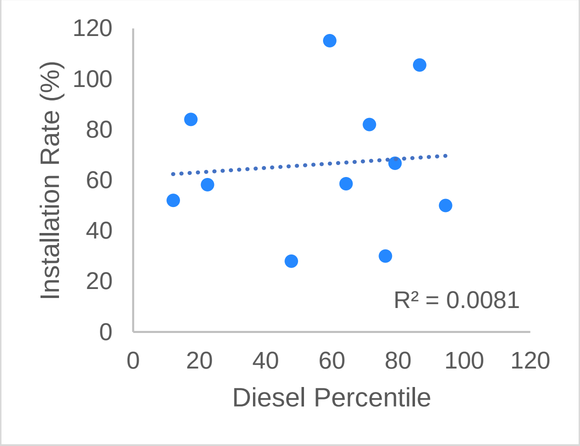

Figure S6 (left) – PM2.5 Percentile Values; Figure S7 (right) – Diesel Percentile Values

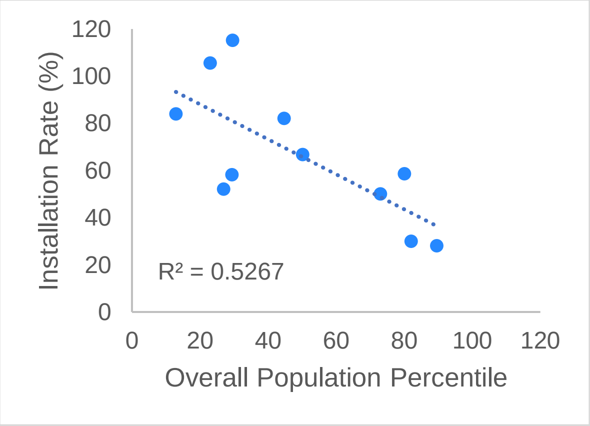

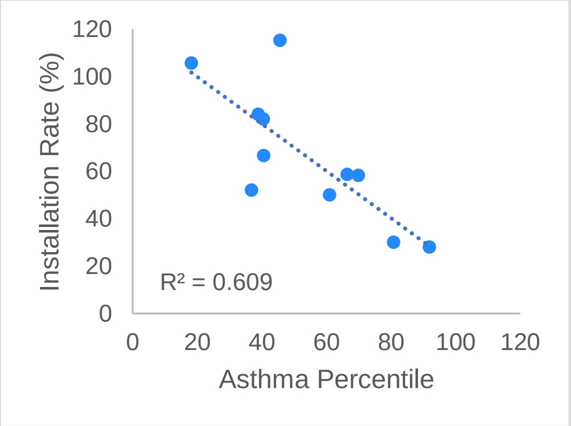

Figure S8 (left) – Overall Population Percentile Values; Figure S9 (right) – Asthma Percentile Values

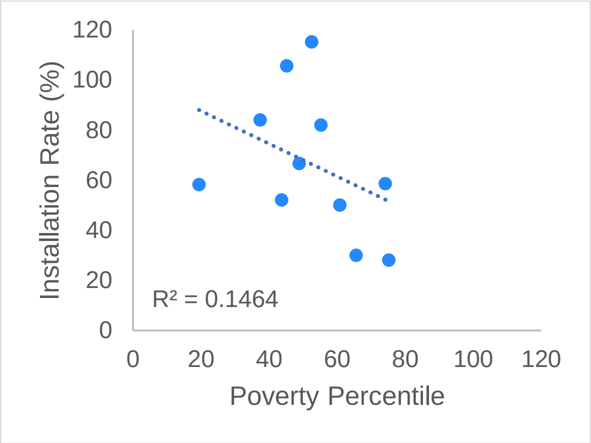

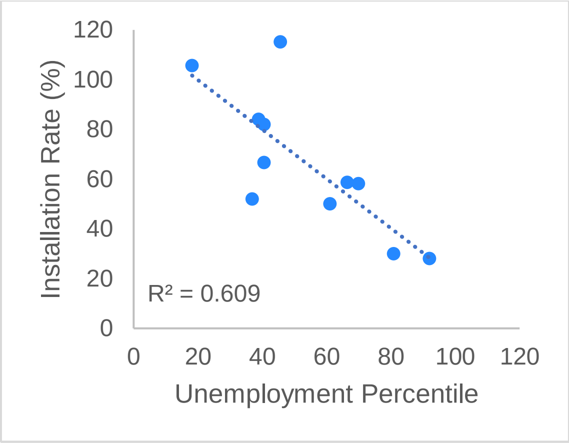

Figure S10 (left) – Poverty Percentile Values; Figure S11 (right) – Unemployment Percentile Values

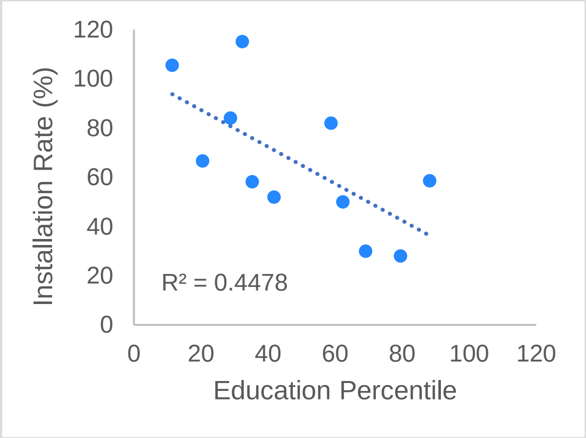

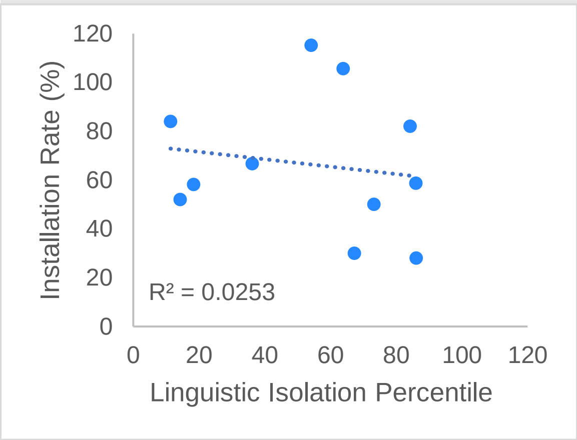

Figure S12 (left) – Education Percentile Values; Figure S13 (right) – Linguistic Isolation Percentile Values

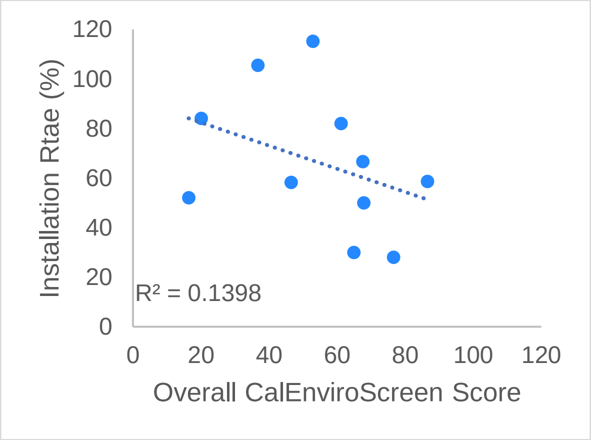

Figure S14 – Overall CalEnviroScreen Score

Photos of Proper and Improper Installations by Project Participants

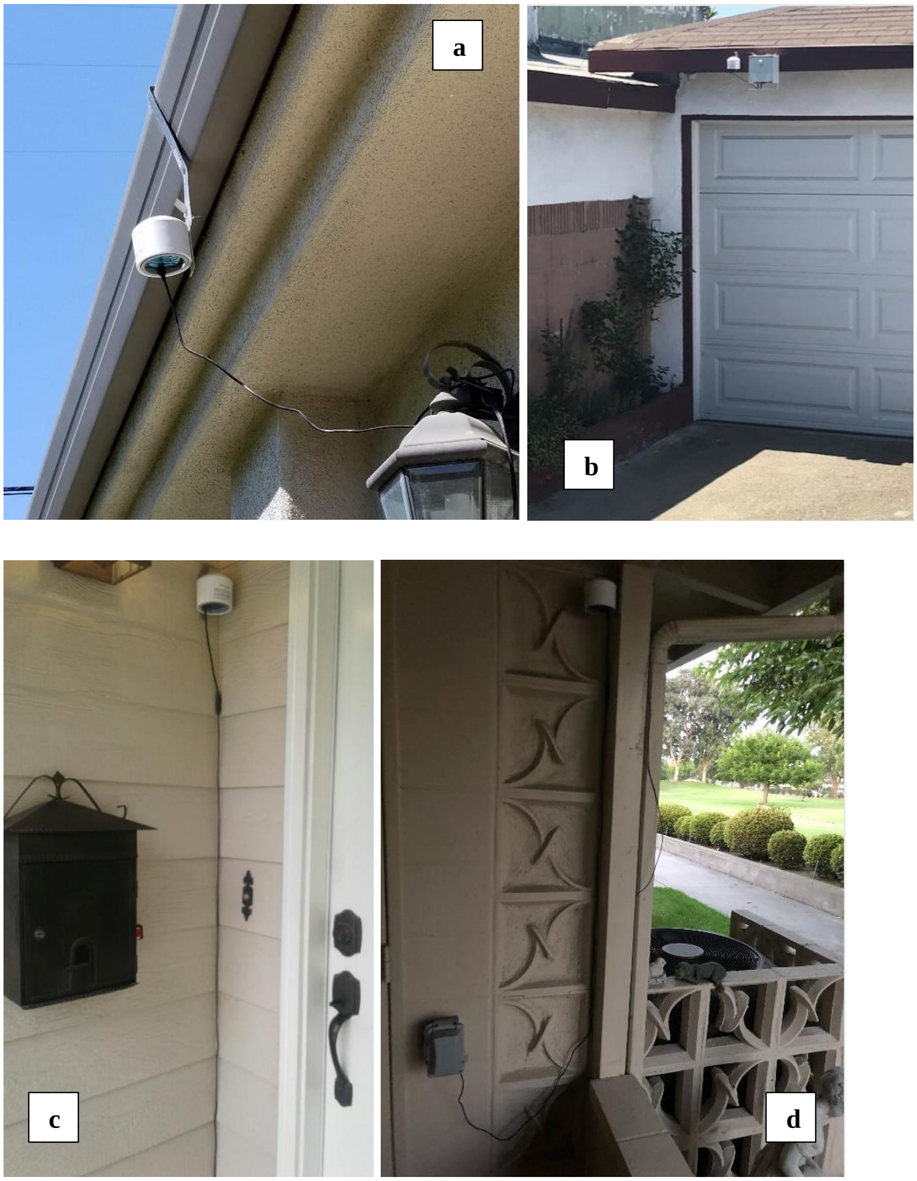

Figure S15 – Here the range of installations by participants are shown. In the top two photos (a and b) the sensors are elevated and have access to free airflow (though the participants would need to note vehicles entering and exiting the garage in the photos on the top-right or b); these are examples of proper installation. In the bottom-left photo (c) the sensor is elevated but is somewhat sheltered and access to airflow is limited. In the bottom-right photo (d) the sensor is above an air conditioning unit that may produce dust and serve as a confounding source, this would be an example of improper siting. While participants from community A submitted most surveys (34 surveys), 45 surveys were submitted by participants in other communities.

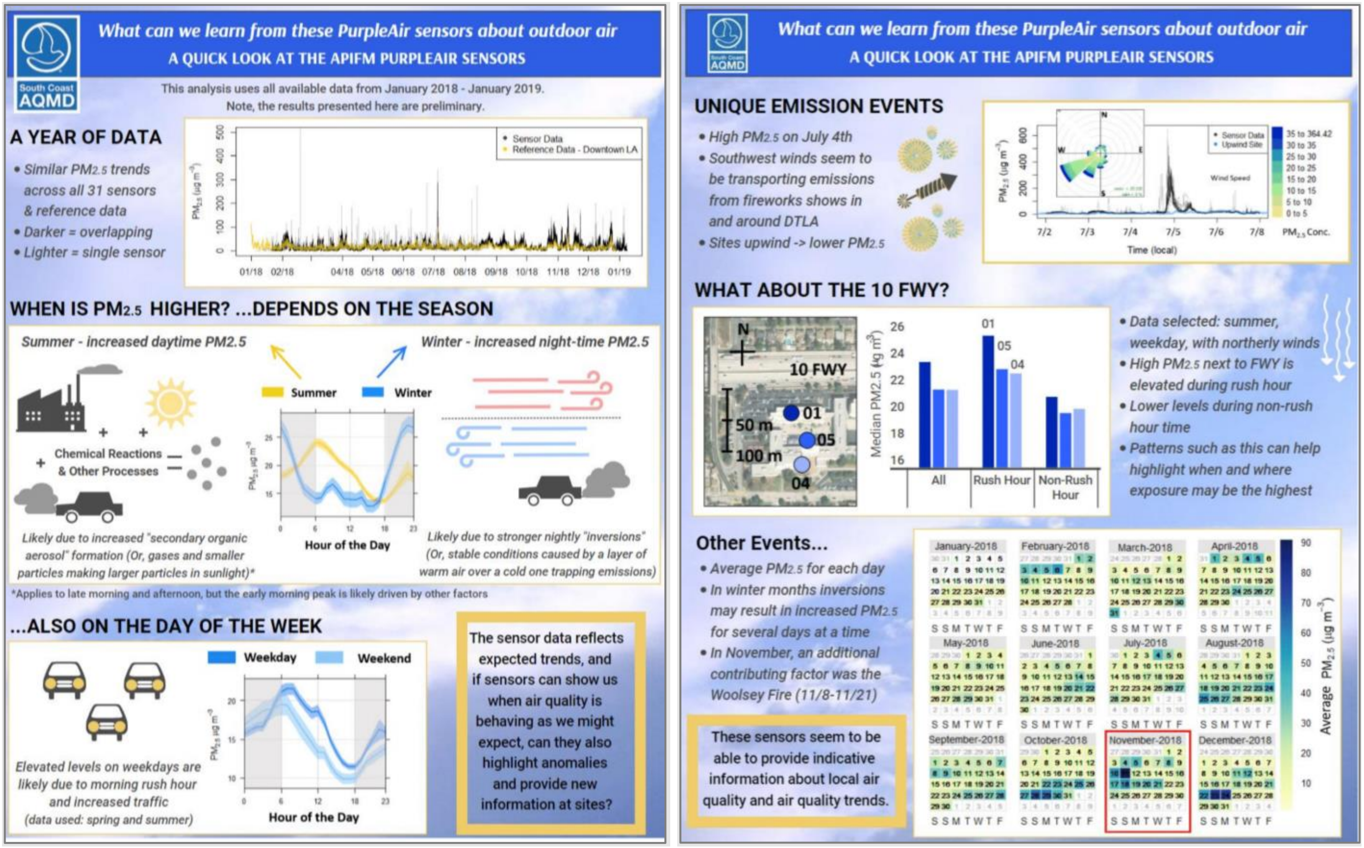

Figure S16 – Example of an infographic designed for and shared with one of the participating communities

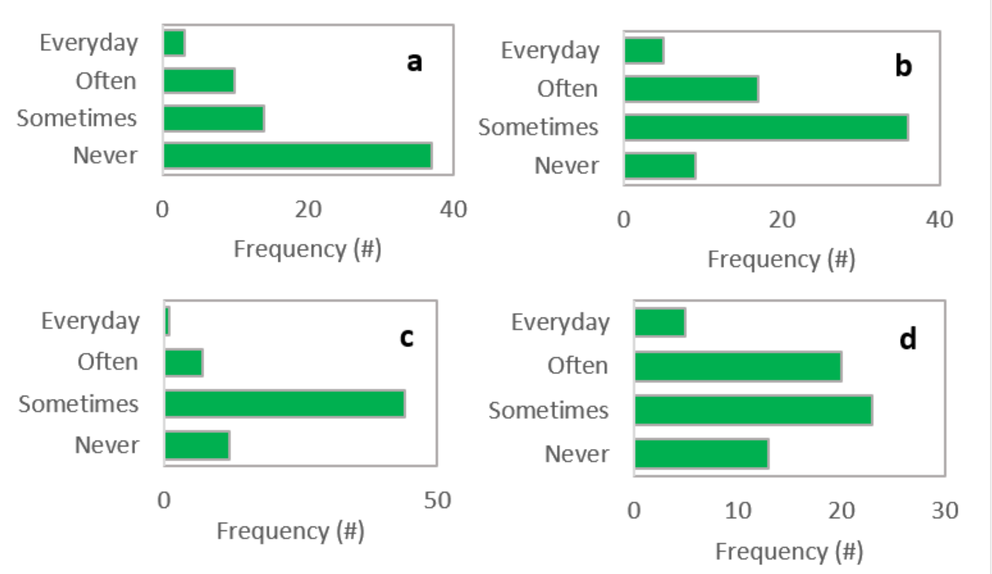

Figure S17: Results from during-the-deployment survey, for the question: (a) “Do you look at other sources of air quality information?”, (b) “Do you regularly look at the low-cost air quality sensor data?”, (c) “Do you have questions about what the data mean?”, and (d) “Have you noticed any relationship between activities and sensor data?” (n = 63)

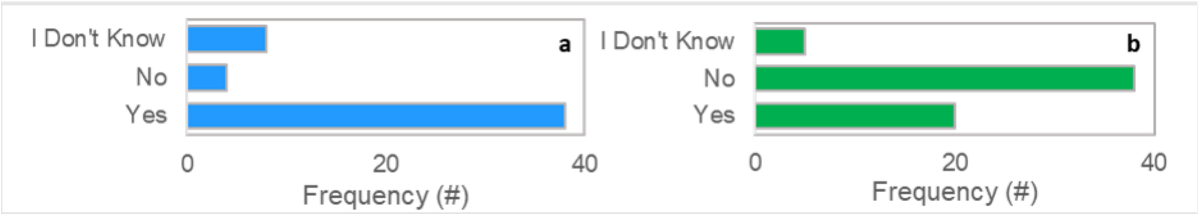

Figure S18: Results from the prior-to-deployment survey, for the question, (a) "Do you think you would change your behavior based on air quality sensor data?", (n = 50) and results from the during-the-deployment survey, for the question, (b) "Have you changed your behavior?" (n = 63)

Examples of Data Analysis and Visualization by Community Participants

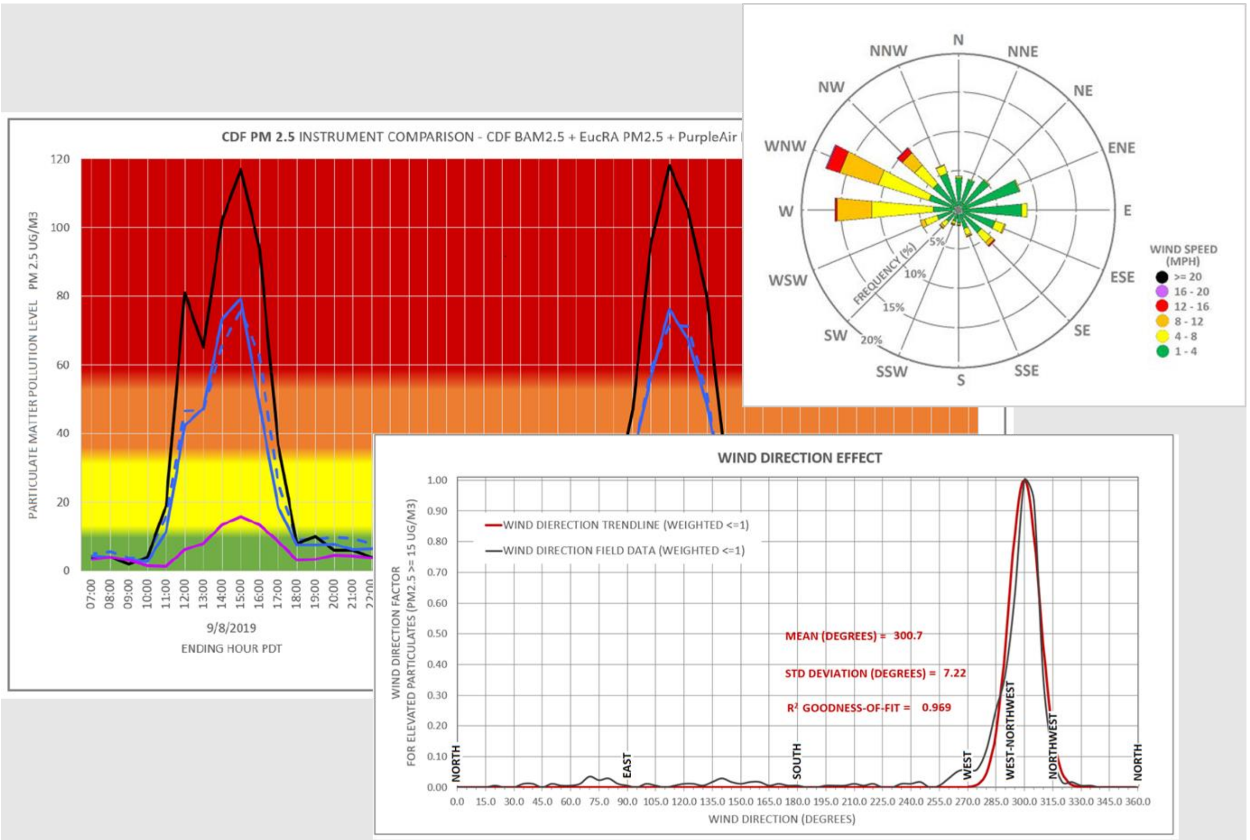

Figures S19 – Examples of data analysis examining sensor performance and the influence of wind speed, conducted by a community member.

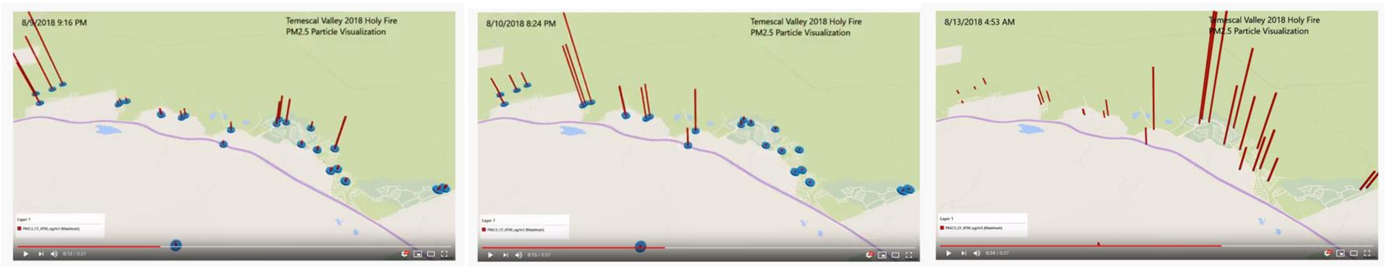

Figure S20 – Screenshot of a data animation showing a wildfire event, the height of the red bars indicated pollutant concentrations, while the size of the blue circle indicates wind speed.

In-Person Survey Statistics

The following statistics refer to the in-person surveys completed during the workshops, the demographics in particular are not necessarily representative of the participant make-up for the entire project.

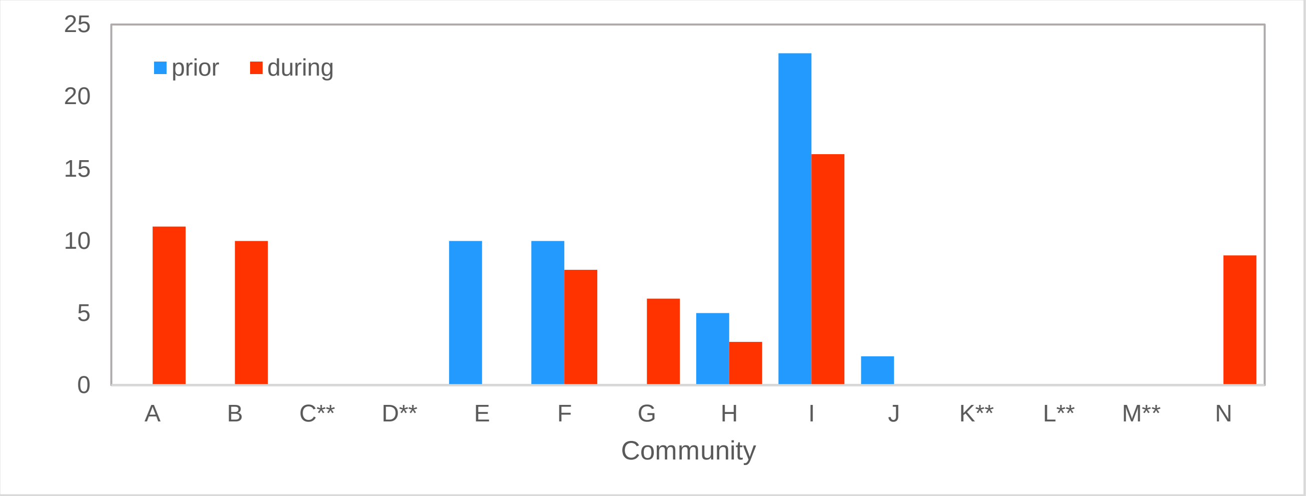

Figure S21 – number of surveys completed prior-to-deployment (in blue) and during-the-deployment (in red) in each community, **denotes communities where either workshops were not held due to the use of a different sensor deployment model or surveys not being distributed/collected by team leading the workshop

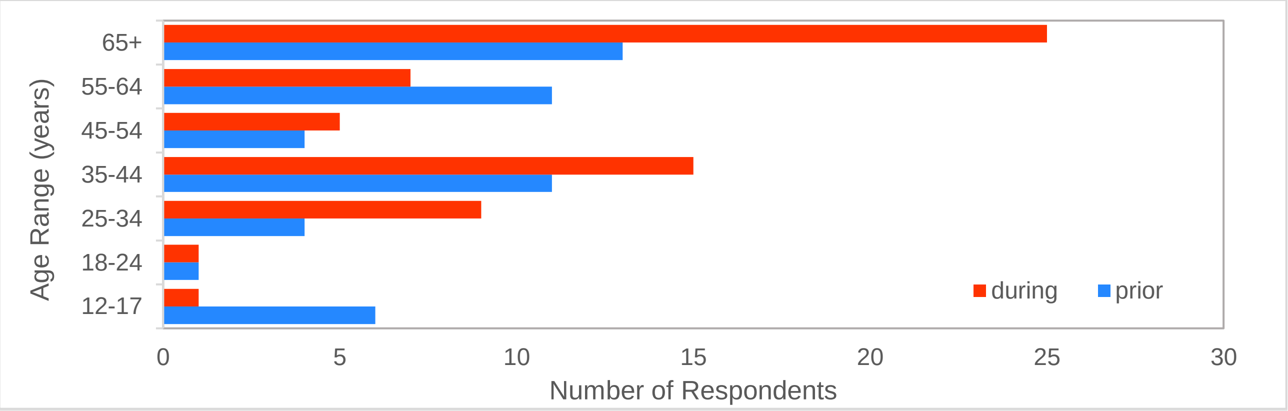

Figure S22 – Aggregate ages of the survey respondents for the prior-to-deployment survey (in blue) and during-the-deployment survey (in red)

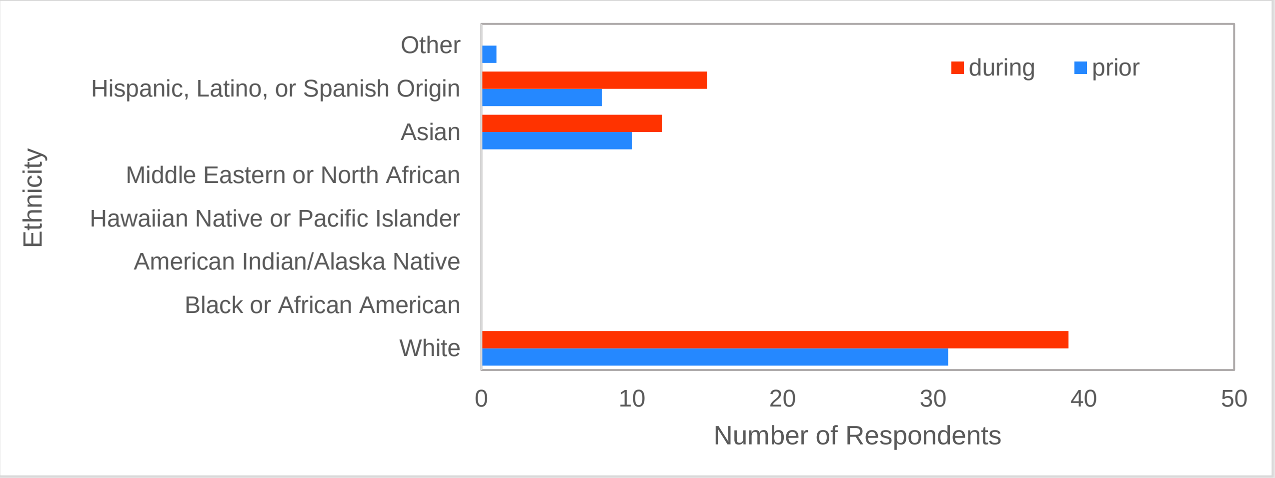

Figure S23 – Aggregate ethnicity of the survey respondents for the prior-to-deployment survey (in blue) and during-the-deployment survey (in red)

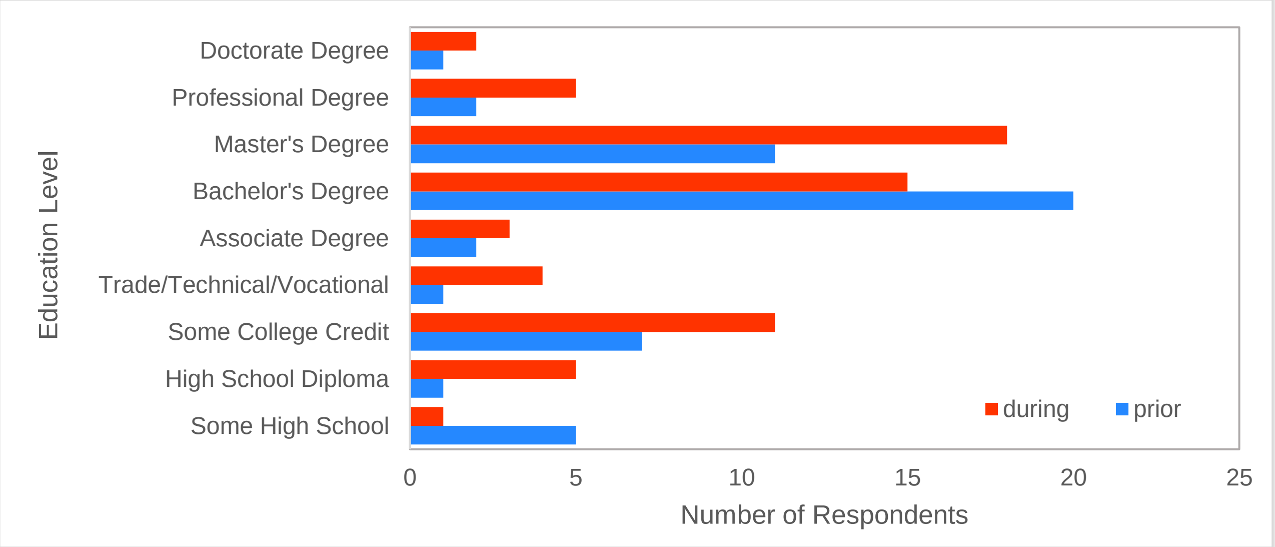

Figure S24 - Aggregate education level of the survey respondents for the prior-to-deployment survey (in blue) and during-the-deployment survey (in red)

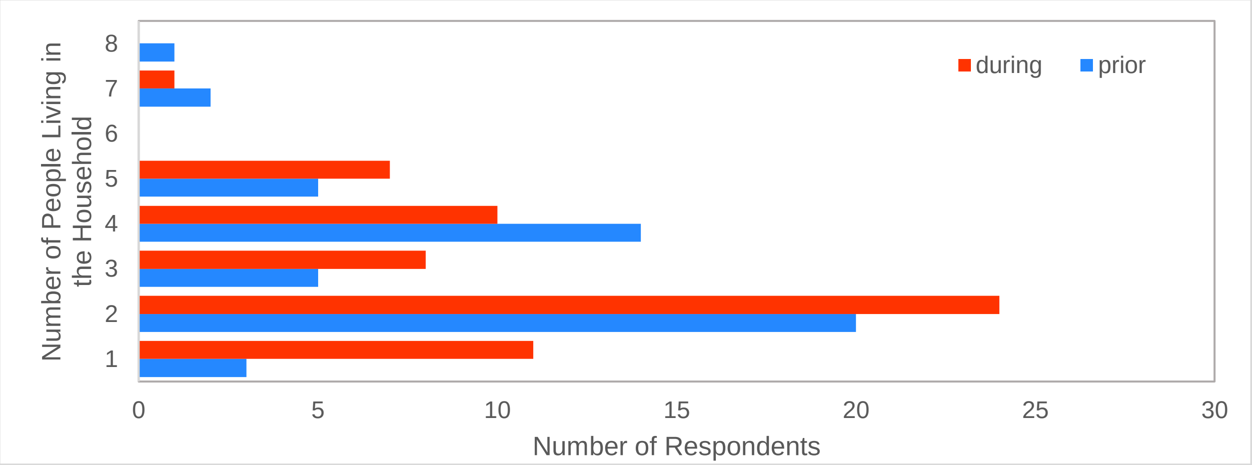

Figure S25 – Number of people living in the home for the prior-to-deployment surveys (in blue) and during-the-deployment surveys (in red)
